# Giant cell tumor of the frontal sinus: a typical finding in an unlikely location

**DOI:** 10.1590/0100-3984.2016.0060

**Published:** 2017

**Authors:** Beatriz Morais e Rodrigues Cunha, Marcelo Fontalvo Martin, João Maurício Canavezi Indiani, Marcelo Souto Nacif

**Affiliations:** 1 Unidade de Radiologia Clínica (URC), São José dos Campos, SP, Brazil; 2 Universidade Federal Fluminense (UFF), Niterói, RJ, Brazil

**Figure 1:**
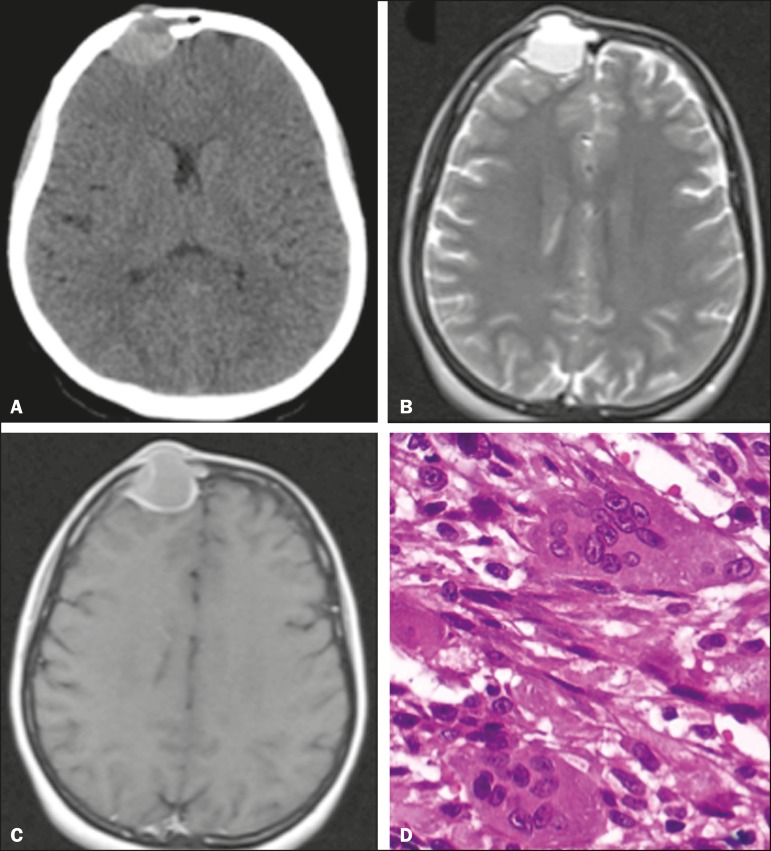
**A:** Axial CT of the skull, after intravenous administration of contrast material, showing a dense, spontaneous, expansile extra-axial formation, measuring 3.1 × 2.5 × 2.9 cm, with its epicenter in the right frontal sinus, featuring bone destruction, an evident air-fluid level, and well-defined borders. **B:** T2-weighted axial MRI slice that best identified the predominantly cystic lesion with an air-fluid level due to the blood content, responsible for the rapid expansion of the tumor. **C:** Contrast-enhanced axial MRI slice showing marked peripheral enhancement. **D:** Histological section stained with hematoxylin and eosin, demonstrating spindle cell morphology, in a fascicular pattern, surrounding numerous large multinucleated osteoclasts.

*Dear Editor*,

A 32-year-old female patient was admitted to the emergency room complaining of a knot on her forehead that had appeared 24 hours earlier. The patient underwent computed tomography (CT) of the skull, with and without intravenous administration of iodinated contrast medium. The CT scans revealed a dense, spontaneous, expansile extra-axial formation with its epicenter in the right frontal sinus, featuring an evident air-fluid level and well-defined borders (Figure 1A). On T2-weighted magnetic resonance imaging (MRI) sequences, the lesion also showed an air-fluid level (Figure 1B). A contrast-enhanced axial MRI scan showed peripheral enhancement (Figure 1C). The patient underwent surgery for complete resection of the lesion. The pathological examination demonstrated tumor-free margins, and immunohistochemistry showed that the lesion was characteristic of a giant cell tumor (GCT) of bone (Figure 1D).

GCT is one of the most common primary bone tumors, accounting for approximately 10% of all bone tumors and 25% of all benign bone tumors^(1)^. It mainly affects individuals 20-40 years of age and has an insidious onset, presenting with pain and a local increase in volume^(1)^. It is usually located in the epiphyses or metaphyses of the long bones, most commonly in the knees (distal femur or proximal tibia). Although it affects less than 1% of all bone sites within the skull (mainly the temporal and sphenoid bones), GCT tends to be more aggressive when it occurs at such sites^(2-4)^.

Based on the classical radiographic aspects, GCT of bone can be defined as a lytic, expansile lesion, resulting in thinning or erosion of the cortical bone^(5)^. CT is the best method to evaluate bone destruction and to identify pathological fractures. MRI can reveal soft tissue invasion and cystic areas (secondary aneurysmal hemorrhages or cysts)^(6,7)^. The definitive diagnosis is made through the identification of giant cells in the histological analysis.

We believe that radiological symptom assessment is of great importance for the diagnosis of bone diseases, because some lesions allow a specific etiological diagnosis, whereas others must be treated on the basis of the description of the findings alone. In the present case, the radiological findings were quite typical. However, the extremely atypical location made it difficult to establish a specific diagnosis. There have been few reported cases of GCT of the skull; hence the relevance of this case.

In the case presented here, CT and MRI were both of extreme importance in the surgical planning and in the postoperative follow-up. The prognosis was favorable, and the patient progressed well in the postoperative period, without the need for radiotherapy. At this writing, she has been followed for approximately two years, without complaints or signs of local recurrence.
